# Long-term recurrence of cholesteatoma after surgery: pooled rates and determinants

**DOI:** 10.1093/bjsopen/zraf131

**Published:** 2025-11-04

**Authors:** Saqr Massoud, Raed Farhat, Uday Abd Elhadi, Bashir Abu Abed, Shlomo Merchavy, Alaa Safia

**Affiliations:** Department of Otolaryngology, Head and Neck Surgery Unit, Ziv Medical Center, Safed, Israel; Department of Otolaryngology, Head and Neck Surgery Unit, Ziv Medical Center, Safed, Israel; Department of Otolaryngology, Head and Neck Surgery Unit, Ziv Medical Center, Safed, Israel; Department of Internal Medicine, Brazilai Medical Center, Ashkelon, Israel; Department of Otolaryngology, Head and Neck Surgery Unit, Ziv Medical Center, Safed, Israel; Department of Otolaryngology, Head and Neck Surgery Unit, Ziv Medical Center, Safed, Israel

Cholesteatoma is a destructive lesion of the middle ear that, despite surgical removal, frequently recurs^1^. Reported recurrence rates vary widely depending on patient age, disease severity, and surgical approach^2^. Although previous systematic reviews^3,4^ have addressed specific aspects of recurrence, none have comprehensively evaluated clinical and surgical predictors across a large global cohort. A meta-analysis was conducted to determine recurrence rates, identify key risk factors, and explore the long-term cumulative incidence rate following cholesteatoma surgery.

This review was registered in PROSPERO (CRD42024550351) and followed PRISMA guidelines. PubMed, Scopus, Web of Science, CENTRAL, and Google Scholar were searched during 2000–2024. Observational studies and randomized trials reporting postoperative recurrence in ≥ 20 patients were eligible. Recidivism was excluded. Data extraction and study selection were performed independently by two investigators, with disagreements resolved by consensus.

Random-effects meta-analysis of recurrence proportions was carried out. Predefined subgroup analyses included age, stage, cholesteatoma type, and surgical technique. Meta-regression was performed using Stata^®^ version 18 (StataCorp, College Station, TX, USA) meta command to examine continuous predictors (for example percentage undergoing staged or revisional surgery, age, follow-up duration). Study quality was assessed using the Newcastle–Ottawa Scale.

Eighty-four studies (12 819 patients) were included; most were retrospective and of low methodological quality (*Table S1*). Definitions of recurrence were reported inconsistently (31 of 84 studies). The pooled recurrence rate was 11 (95% confidence interval 9 to 13)%, with higher rates in children than adults (13 *versus* 10%). Acquired cholesteatoma recurred more often than the congenital type (12 *versus* 7%). The surgical approach was the strongest determinant. Canal wall down (CWD) procedures had a pooled recurrence rate of 7%, compared with 16% after canal wall up (CWU). Within subtypes, CWU tympanoplasty/tympanomastoidectomy was associated with the highest recurrence rate, whereas CWD tympanoplasty/tympanomastoidectomy had the lowest (19 *versus* 1%). Adjunctive techniques were protective; mastoid obliteration reduced the recurrence rate to 9% compared with 29% without obliteration, and complete ossicular reconstruction reduced the rate to 6% compared with 16% with no reconstruction. Cumulative analysis revealed that the recurrence rate increased with time: 7% at 12 months, 18% at 60 months, and up to 39% at 15 years, highlighting that short follow-up times markedly underestimated disease burden (*Fig. 1*). In adjusted meta-regression analysis, increasing age (β = 0.0036, *P* = 0.006), staged surgery (β = 0.0153, *P* = 0.029), and planned second-look surgery (protective; β = −0.0145, *P* = 0.030) independently predicted recurrence.

**Fig. 1:**
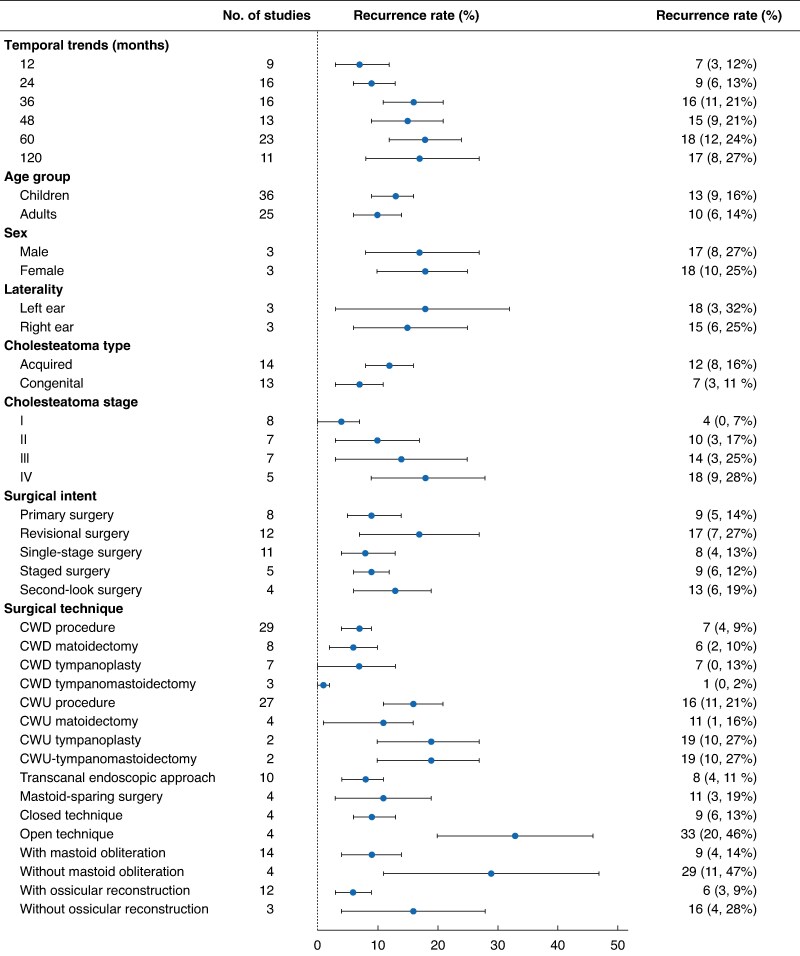
Pooled recurrence rates of cholesteatoma following surgical management, stratified by follow-up duration, age, cholesteatoma type, stage, surgical intent, and surgical technique CWD, canal wall down; CWU, canal wall up.

This meta-analysis demonstrated that approximately one in ten patients experience recurrent cholesteatoma after surgery, with the risk continuing to rise over long-term follow-up. CWD procedures, mastoid obliteration, ossicular reconstruction, and proactive second-look operations were consistently associated with lower recurrence risk. Conversely, CWU approaches and advanced-stage disease carried a higher risk. These findings are clinically relevant for surgical decision-making and patient counselling. In particular, the sharp increase in recurrence beyond 5 years underscores the importance of long-term surveillance, whether by second-look surgery or diffusion-weighted magnetic resonance imaging^5^.

The results must be interpreted with caution. More than 90% of included studies were of low methodological quality, and heterogeneity was substantial. Most studies did not clearly distinguish residual from true recurrent disease, a limitation that future prospective studies should address by use of standardized definitions. Other relevant outcomes, such as hearing, complications, and quality of life, were not assessed here; integrating these alongside recurrence would provide a more balanced appraisal of surgical strategies.

In conclusion, recurrence after cholesteatoma surgery remained common, rising to nearly 40% with long-term follow-up. CWD procedures with obliteration and ossicular reconstruction reduced recurrence risk, whereas staged surgery and paediatric disease conferred a higher risk. Standardized definitions and prospective long-term studies are needed to refine surgical strategies and surveillance protocols.

## Supplementary Material

zraf131_Supplementary_Data

## Data Availability

The data sets used and/or analysed during the present study are available from the corresponding author on reasonable request.
