# Supplementary material for: Long-term recurrence of cholesteatoma after surgery: pooled rates and determinants
Source: BJS Open. 2025 Nov 4;9(6):zraf131. doi: 10.1093/bjsopen/zraf131 (PMC12587149; doi:10.1093/bjsopen/zraf131)
Supplement: zraf131_Supplementary_Data [file zraf131_supplementary_data.docx]

**Long-term recurrence of cholesteatoma after surgery: pooled rates and determinants**

Saqr Massoud^1^, Raed Farhat^1^, Uday Abd Elhadi^1^, Bashir Abu Abed^2^, Shlomo Merchavy^1^, Alaa Safia^1,*^

^1^Department of Otoloaryngology, Head & Neck Surgery Unit, Rebecca Ziv Medical Center, Safed, Israel

^2^General doctor, Brazilai Medical Center, Ashkelon, Israel

**^*^ Corresponding author.** Alaa Safia, Department of Otolaryngology, Head & Neck Surgery Unit, Rebecca Ziv Medical Center, Derech HaRambam 1, Safed, Israel. Telephone number: +972 50-575-0787. (email: [alaa.safia03@gmail.com](mailto:alaa.safia03@gmail.com)). **ORCID ID: 0000-0002-9574-8729**.

**Supplementary Materials - Index**

| **Supplementary Figures and Tables** |  |
| --- | --- |
| Table S1. Baseline characteristics | *page 2* |
| **References** | *page 6* |
|  |  |

**Table S1.** Baseline characteristics

| Author (YOP) | Design | Country | YOI | Mean Follow-up | Sample Size | | Age | | Gender | | Surgery |
| --- | --- | --- | --- | --- | --- | --- | --- | --- | --- | --- | --- |
|  |  |  |  |  | Patients | Ears | Total Population | | Total | |  |
|  |  |  |  |  |  |  | Mean | SD | Male | Female |  |
| Abdullah et al. (2013) ^1^ | Retrospective Study | Malaysia | June 1996 - December 2003 | - | 63 | 63 | 31 years | 5 mo - 72 years | 26 | 37 | CWDM |
| Abraham et al. (2022) ^2^ | Prospective observational study | India | September 2017 to August 2018 | 6 | 380 | 380 | 31.3 | (18 - 62) | - | - | CWUM + CWDM |
| Adriaansens et al. (2022) ^3^ | Retrospective Study | The Netherlands | 2015 and 2018 | 35 | 67 | 67 | 40 | (5-79) | 44 | 23 | Combined approach T |
| Alam (2022) ^4^ | Comparative Prospective study | India | January 2016 to June 2020 | 32 | 100 | 100 | - | - | - | - | CWUTM + CWDTM |
| Alicandri-Ciufelli et al. (2016) ^5^ | Prospective Study | Italy | January 2006 to September 2015 | 36 | 234 | 244 | - | - | - | - | Endoscopic management |
| Alvarez et al. (2011) ^6^ | Retrospective Study | Spain | July 1977 to September 2007 | 123.6 | 35 | 35 | 54 | (29-77) | 16 | 19 | Open vs. obliterative |
| Arias Marzán et al. (2023) ^7^ | Cohort Study | Spain | 2001 - 2020 | 228 | 65 | 65 | - | - | - | - | Attic Exposition-Antrum Exclusion vs. CWUTM |
| Aslan Felek et al. (2009) ^8^ | Retrospective Study | Turkey | January 1996 - May 2007 | 46.3 | 134 | 134 | 30.1 | 11.7 | 66 | 68 | CWDT |
| Bakaj et al. (2016) ^9^ | Prospective Study | Czech Republic | 2012 | 72 | 24 | 24 | 34 | (9-63) | 13 | 11 | CWDM + CWUM |
| Barakate (2008) ^10^ | Retrospective Study | United Kingdom | January 1998 - December 2004 | 18 | 66 | 66 | 18 | (5-63) | - | - | Combined approach T |
| Chamoli et al. (2018) ^11^ | Retrospective Study | Denmark | January 2002 to December 2005 | 120 | 178 | 178 | (<15 = 77) | (>15 = 101) | 87 | 91 | CWU, CWD, CWR, non-mastoidectomy |
| Cheng et al. (2023) ^12^ | Retrospective Study | Taiwan | October 2007 to December 2021 | 77 | 24 | 24 | 4.8 | (1.3-9.8) | 18 | 6 | Exploratory tympanotomy, CWUM, Transcanal attictomy |
| Cho et al. (2016) ^13^ | Retrospective Study | Republic of Korea | January 1997 - June 2012 | 12 | 93 | 93 | 6.1 | (1 - 17) | 64 | 29 | Transcanal approach, CWUM, Tympanoplasty, CWDM |
| Choi et al. (2010) ^14^ | Retrospective Study | Republic of Korea | 1997 - 2007 | 132 | 71 | 71 | 5.6 | (1-15) | 52 | 19 | Tympanoplasty, TE (T+Epitympanoplasty), CWU, CWD, Ossiculoplasty |
| Crowson et al. (2016) ^15^ | Retrospective Study | USA | 2009 - 2014 | 43.75 | 106 | 106 | 36.5 | (2-90) | 65 | 76 | CWUTM |
| Danesi et al. (2016) ^16^ | Retrospective Study | Italy | 1989 - 2009 | 240 | 81 | 81 | 46.9 | 14.6 | 57 | 24 | - |
| Darrouzet et al. (2000) ^17^ | Retrospective Study | France | 1985 - 1996 | 70 | 215 | 215 | 9.6 | (1-15) | 114 | 101 | RM or CT or TOT |
| Das et al. (2019) ^18^ | Retrospective Study | India | March 2016 - February 2018 | 60 | 32 | 32 | 33.27 | (10-55) | 14 | 18 | RM and Modified RM |
| De Corso et al. (2006) ^19^ | Retrospective Study | Italy | January 1992 - December 2002 | 60 | 368 | 368 | (11 / 45) | (Children = 4-16, Adults = 17-75) | 198 | 170 | CWDT |
| DeRowe et al. (2005) ^20^ | Retrospective Study | Israel | 1979 - 1994 | 61.2 | 53 | 53 | 10.7 | 3.3 | 34 | 17 | Atticotomy |
| Diom et al. (2013) ^21^ | Retrospective Study | Senegal | 01 Jan 1995 to 31 Dec 2009 | 60 | 66 | 66 | 10 | (2-15) | 39 | 27 | RM + Modified RM |
| Edfeldt et al. (2012) ^22^ | Retrospective Study | Sweden | 1983 - 2004 | 72 | 57 | 57 | 8.2 | (2.3-11.9) | - | - | CWD + total reconstruction procedure (TRP) |
| Edfeldt et al. (2013) ^23^ | Retrospective Study | Sweden | 1982 - 2004 | 72 | 330 | 330 | 38 | (2-83) | 195 | 135 | CWD + TRP |
| Erfurt et al. (2024) ^24^ | Retrospective Study | Netherlands | Jan 2015 - March 2020 | 24 | 143 | 143 | 43.5 | (18-84) | 88 | 55 | CWUTM |
| Ferlito et al. (2022) ^25^ | Retrospective Study | Italy | Jan 2002 - Dec 2022 | 120 | 176 | 176 | 46.41 | 10.1 | 105 | 71 | CWDT |
| Glikson et al. (2019) ^26^ | Retrospective Study | Israel | March 2009 - March 2016 | 34.9 | 32 | 32 | - | - | 25 | 17 | TEA + CWUTM |
| Govil et al. (2015) ^27^ | Retrospective Study | United States | Jan 1, 2000 - Dec 31, 2012 | 33.6 | 55 | 55 | - | - | - | - | - |
| Hatano et al. (2010) ^28^ | Retrospective Study | Japan | October 2002 and August 2008 | 48.7 | 25 | 25 | 9 | (4-15) | 22 | 3 | Retrograde M |
| Hatano et al. (2016) ^29^ | Retrospective Study | Japan | October 2002 and August 2008 | 99 | 24 | 25 | - | - | 21 | 3 | Retrograde M |
| Hellingman et al. (2019) ^30^ | Retrospective Study | Netherlands | 2010 to 2014 | 39.6 | 96 | 99 | - | - | - | - | CWU |
| Hou et al. (2021) ^31^ | Retrospective Study | China | Jan 2017 and December 2017 | 24 | 34 | 34 | 37 | (18-64) | 23 | 11 | TEA |
| Hu et al. (2023) ^32^ | Prospective Study | China | - | 24 | 32 | 32 | - | - | - | - | CWUTM |
| Ikeda et al. (2003) ^33^ | Retrospective Study | Japan | 1990 - 1997 | 24 | 101 | 103 | 43 | (5-75) | 60 | 43 | CWDT |
| Inanli et al. (2001) ^34^ | Retrospective Study | Turkey | 1990 - 1999 | 57 | 347 | 347 | 30 | (4-56) | 40 | 26 | RM, MRM, WCDM, iCWM |
| James (2024) ^35^ | Retrospective Study | Canada | 2005 - 2020 | 55.2 | 408 | 408 | 10.7 | (1.8 - 17.8) | - | - | CWUTM, CWD/MO, no mastoidectomy, TEA |
| Jenks et al. (2022) ^36^ | Retrospective Study | USA | Jan 1, 2011 - Sep 1, 2019 | 46.8 | 64 | 65 | 6.5 | (1.2 - 16) | 46 | 19 | TEA |
| Killeen et al. (2019) ^37^ | Retrospective Study | United States | May 2014 and Sep 2016 | 30 | 59 | 59 | - | - | - | - | TEA, Microscopic Mx |
| Kim et al. (2009) ^38^ | Retrospective Study | Republic of Korea | Oct 2002 and Dec 2006 | 27.4 | 98 | 98 | 41 | (5-65) | 40 | 58 | Atticoantrotomy |
| Komori et al. (2018) ^39^ | Retrospective Study | Japan | Dec 1995 to Oct 2011 | 60 | 115 | 115 | - | - | 62 | 53 | Modified CWUT |
| Komori et al. (2021) ^40^ | Prospective Study | Japan | 1 Jan 2016 - 29 Feb 2016 | 23.4 | 1787 | 1787 | - | - | - | - | - |
| Kuo et al. (2012) ^41^ | Retrospective Study | Taiwan | 1982 - 2011 | 300 | 71 | 71 | 10.7 | - | 42 | 31 | - |
| Lazard et al. (2007) ^42^ | Retrospective Study | France | 1996 - 2005 | 30 | 117 | 117 | 6.5 | (2.7 - 19.0) | 75 | 42 | - |
| Lee et al. (2015) ^43^ | Retrospective Study | South Korea | Jan 2009 - Dec 2014 | - | 24 | 24 | - | - | 18 | 6 | Transcanal Co2 laser-enabled ablation |
| Manzoor et al. (2022) ^44^ | Retrospective Study | USA | 2012 - 2017 | - | 375 | 375 | - | - | - | - | Microscopic and Endoscopic surgery |
| Marchioni et al. (2013) ^45^ | Retrospective Study | Italy | Jan 2006 - May 2012 | 12 | 146 | 146 | - | - | - | - | TEA |
| Marchioni et al. (2015) ^46^ | Retrospective Study | Italy | Jan 2007 and Dec 2013 | 36 | 54 | 59 | 9.6 | (4-16) | 34 | 20 | TEA, CWU |
| Minovi et al. (2014) ^47^ | Retrospective Study | Germany | Jan 2006 and June 2011 | 60 | 218 | 242 | 48.2 | (18-82) | 130 | 88 | Retrograde Mastoidectomy |
| Mishiro et al. (2008) ^48^ | Retrospective Study | Japan | 1987 - 2002 | 60 | 345 | 345 | 41.5 | (1-78) | 177 | 168 | CWDT, iCWT +/- CWR |
| Mizutari et al. (2021) ^49^ | Retrospective Study | Japan | Jan 2015 - Dec 2017 | 35.8 | 74 | 74 | 48.9 | 18.7 | 38 | 36 | TEA |
| Moller et al. (2020) ^50^ | Retrospective Study | Denmark | Jan 1, 1983 and Dec 31, 2015 | 60 | 1006 | 1006 | - | - | 632 | 374 | No mastoidectomy, CWUM +/- MO, and CWDM |
| Morita et al. (2014) ^51^ | Retrospective Study | Japan | 1999 - 2012 | 54.3 | 67 | 67 | - | - | 52 | 15 | CWDT + MO (adults), TCA (children) |
| Morita et al. (2017) ^52^ | Retrospective Study | Japan | Jan 1999 - Dec 1999 | 62 | 42 | 42 | 9.4 | (3-15) | 38 | 4 | CWUTM, TCA |
| Motegi et al. (2020) ^53^ | Retrospective Study | Japan | Jan 2009 - Dec 2015 | 81.7 | 127 | 127 | 42.6 | (6-83) | 73 | 54 | iCWT |
| Myers et al. (2000) ^54^ | Retrospective Study | Denmark | Jan 1980 - Dec 1994 | 96 | 33 | 33 | - | - | - | - | - |
| Nassif et al. (2024) ^55^ | Retrospective Study | Italy | Jan 2010 - Dec 2020 | 144 | 71 | 71 | 9 | (3-16) | 50 | 21 | CWDTM, CWUTM, TEA |
| Neudert et al. (2014) ^56^ | Retrospective Study | Germany | 1994 untill Nov 2010 | 34.8 | 406 | 116 | 42.2 | 21.3 (3.1 - 89.8) | 206 | 200 | ETA, TCM, iCWT, CWD |
| Pareschi et al. (2019) ^57^ | Retrospective Study | Italy | Jan 1992 and March 2016 | 120 | 895 | 895 | - | - | - | - | CWDTM |
| Park et al. (2009) ^58^ | Retrospective Study | Republic of Korea | 1995 - 2006 | 47 | 35 | 35 | 6.2 | (2-13) | 27 | 8 | T, ET, CWUM, CWDM |
| Park et al. (2011) ^59^ | Retrospective Study | Republic of Korea | Jan 1990 and August 2007 | 120 | 42 | 42 | 47 | (24-73) | - | - | CWUTM, CWDTM |
| Piras et al. (2021) ^60^ | Retrospective Study | Italy | 1983 - 2015 | 48 | 236 | 236 | 10.8 | 3.3 | 151 | 73 | CWUTM, CWDTM |
| Prasad et al. (2014) ^61^ | Retrospective Study | Italy | 2001 - 2005 | 60 | 40 | 40 | 10.1 | (2-16) | 24 | 16 | iCWT |
| Presutti et al. (2018) ^62^ | Retrospective Study | Italy | - | 29.7 | 110 | 110 | 37.1 | (8-78) | - | - | TEA, CWU |
| Qotb et al. (2017) ^63^ | Retrospective Study | Egypt | 2009 - 2012 | 60 | 64 | 71 | 33 | 7.5 | 41 | 23 | CWDM |
| Quérat et al. (2014) ^64^ | Retrospective Study | France | 2003 - 2012 | 24 | 128 | 128 | 35.5 | (6-77) | 69 | 59 | CWUT |
| Reddy et al. (2001) ^65^ | Retrospective Study | England | 1991 - 1997 | 72 | 103 | 103 | - | - | 55 | 48 | Transcanal Atticoaditotomy and Transcanal Mastoidectomy |
| Roth et al. (2013) ^66^ | Retrospective Study | Switzerland | 1992 - 2008 | 102 | 126 | 130 | 10.5 | (3-16) | 83 | 43 | Inside-out technique T |
| Roux et al. (2015) ^67^ | Retrospective Study | France | May 2008 - May 2012 | 24 | 39 | 36 | 46 | 16 | 24 | 12 | CWUTM |
| Schraff (2006) ^68^ | Retrospective Study | USA | 1 July 1992 - 1 July 2003 | 60 | 262 | 262 | 9.2 | (3-17) | 170 | 92 | iCWT, CWD |
| Shin et al. (2023) ^69^ | Retrospective Study | Korea | August 1993 - January 2016 | 47 | 186 | 186 | 4.9 | 2.8 | 136 | 50 | Transcanal removal, iCWM, open cavity M |
| Silvola (2000) ^70^ | Prospective Study | Finland | 1974 - 1986 | 51.6 | 42 | 42 | 11.6 | (6.5-16) | 31 | 11 | CWD, CWU |
| Song et al. (2019) ^71^ | Retrospective Study | Republic of Korea | August 2007 and November 2014 | 30 | 38 | 38 | 7.9 | 8.2 | 20 | 18 | TM, M, T, removal (not specified) |
| Sun et al. (2010) ^72^ | Retrospective Study | China | Jan 1999 to Dec 2006 | 36 | 45 | 48 | 10 | (5-12) | 33 | 12 | CWDM |
| Trinidade et al. (2015) ^73^ | Prospective Study | United Kingdom | 1999 - 2013 | 60 | 55 | 58 | 10.8 | - | 42 | 16 | CWD |
| van Dinther et al. (2015) ^74^ | Case series | The Netherlands | Sep 1997 - Aug 2009 | 60 | 33 | 34 | 12.6 | (6-18.5) | 21 | 13 | CWU-BOT |
| van Waegeningh et al. (2021) ^75^ | Retrospective Study | Belgium | 2009 - 2014 | 45 | 60 | 61 | - | - | - | - | CWD |
| Vartiainen (2000) ^76^ | Retrospective Study | Finland | 1976 - 1988 | 120 | 136 | 136 | - | - | 54 | 82 | CWDM |
| Visvanathan et al. (2012) ^77^ | Retrospective Study | United Kingdom | 1999 - 2009 | 120 | 99 | 54 | 9.5 | (2-18) | 46 | 53 | - |
| Walker et al. (2014) ^78^ | Retrospective Study | USA | 1997 - 2011 | 51.48 | 273 | 285 | 35 | 18.6 | 175 | 98 | CWRTM |
| Wilson et al. (2013) ^79^ | Retrospective Study | USA | 1998 - 2006 | 63.6 | 148 | 156 | 27 | (3-77) | 96 | 52 | iCWTM |
| Wu et al. (2020) ^80^ | Prospective Study | China | June 2016 to December 2017 | 24 | 57 | 57 | - | - | 36 | 21 | EES |
| Yamamoto et al. (2014) ^81^ | Retrospective Study | Japan | - | 60 | 118 | 113 | 48.4 | - | 60 | 58 | CWDT |
| Yang et (2014) ^82^ | Prospective Study | United Kingdom | 1994 - 2009 | 65.5 | 37 | 37 | 38 | (11 - 67) | 20 | 17 | CWDM |
| Yung et al. (2007) ^83^ | Retrospective Study | United Kingdom | 1988 - 2001 | 60 | 50 | 50 | - | (5-16) | 29 | 21 | T, iCWM, CWDM |
| Zanetti et al. (2018) ^84^ | Retrospective Study | Italy | Jan 2000 and Dec 2012 | 84 | 16 | 20 | 10.9 | (7-18) | - | - | CWUM, CWD |

YOP: year of publication; YOI: year of investigation; USA: United States of America; CWU: canal wall-up; CWD: canal wall-down; CWUM: canal wall-up mastoidectomy; CWUT: canal wall-up tympanoplasty; CWUTM: canal wall-up tympanomastoidectomy; CWDM: canal wall-down mastoidectomy; CWDT: canal wall-down tympanoplasty; CWDTM: canal wall-down tympanomastoidectomy; CWR: canal wall reconstruction; EES: endoscopic ear surgery; T: tympanoplasty; CAT: combined approach tympanoplasty; TM: tympanomastoidectomy; M: mastoidectomy; iCWT: intact canal wall tympanoplasty; TEA: transcanal endoscopic approach; MO: mastoid obliteration.

**References**

1. Abdullah ABH, S. M. Awang, M. A. Saim, L. Outcome of canal wall down mastoidectomy: Experience in Sixty three cases. *Medical Journal of Malaysia* 2013;**68**(3): 217-221.

2. Abraham LP, A. Lepcha, A. Augustine, A. M. Mathews, S. S. Paul, R. R. Mammen, M. D. A Comparative Study of Outcomes and Quality of Life in Canal Wall up Mastoidectomies and Canal Wall down Mastoidectomies. *Indian Journal of Otolaryngology and Head and Neck Surgery* 2022;**74**: 600-607.

3. Adriaansens CB, S Aarts, MCJ. Determinants influencing cholesteatoma recurrence in daily practice: a retrospective analysis. *The Journal of Laryngology & Otology* 2022;**136**(2): 119-124.

4. Alam MC, K. Ears with Cholesteatoma: Outcomes of Canal Wall Up and Down Tympano-Mastoidectomies-A Comparative Prospective Study. *Indian journal of otolaryngology and head and neck surgery : official publication of the Association of Otolaryngologists of India* 2022;**74**(Suppl 1): 730-736.

5. Alicandri-Ciufelli MM, D. Kakehata, S. Presutti, L. Villari, D. Endoscopic Management of Attic Cholesteatoma Long-Term Results. *OTOLARYNGOLOGIC CLINICS OF NORTH AMERICA* 2016;**49**(5): 1265-+.

6. Alvarez FLG, J. R. Bernardo, M. J. Suárez, C. Management of petrous bone cholesteatoma: open versus obliterative techniques. *European archives of oto-rhino-laryngology : official journal of the European Federation of Oto-Rhino-Laryngological Societies (EUFOS) : affiliated with the German Society for Oto-Rhino-Laryngology - Head and Neck Surgery* 2011;**268**(1): 67-72.

7. Arias Marzán FPC, E. R. Lemes Robayna, A. Salom Lucena, M. C. De Lucas Carmona, G. Muñoz Cordero, M. G. Macias Rodríguez, D. H. Jimenez Sosa, A. Effectiveness and Safety of Attic Exposition–Antrum Exclusion versus Canal Wall-Up in Patients with Acquired Stage Ib and II Cholesteatoma Affecting the Attic and Tympanic Cavity. *Journal of Clinical Medicine* 2023;**12**(1).

8. Aslan Felek SI, Ahmet Celik, Hatice Demirci, Munir Samim, Erdal Kose, S Kenan. The functional and anatomical results of the canal wall down tympanoplasty in extensive cholesteatoma. *Acta oto-laryngologica* 2009;**129**(12): 1388-1394.

9. Bakaj TBZ, L. Salzman, R. Tedla, M. Starek, I. Recidivous cholesteatoma: DWI MR after canal wall up and canal wall down mastoidectomy. *Bratislava Medical Journal* 2016;**117**(9): 515-520.

10. Barakate MB, I. Combined approach tympanoplasty for cholesteatoma: impact of middle-ear endoscopy. *The Journal of laryngology and otology* 2008;**122**(2): 120-124.

11. Chamoli PS, C. V. Radia, S. Shah, A. K. Functional and Anatomical Outcome of Inside Out Technique For Cholesteatoma Surgery. *Am J Otolaryngol* 2018;**39**(4): 423-430.

12. Cheng TCH, C. Y. Wu, J. L. The role of planned two-stage surgery in the management of congenital cholesteatoma. *INTERNATIONAL JOURNAL OF PEDIATRIC OTORHINOLARYNGOLOGY* 2023;**171**.

13. Cho HSK, H. G. Jung, D. J. Jang, J. H. Lee, S. H. Lee, K. Y. Clinical Aspects and Surgical Outcomes of Congenital Cholesteatoma in 93 Children: Increasing Trends of Congenital Cholesteatoma from 1997 through 2012. *Journal of audiology & otology* 2016;**20**(3): 168-173.

14. Choi HGP, K. H. Park, S. N. Jun, B. C. Lee, D. H. Park, Y. S. Chang, K. H. Park, S. Y. Noh, H. Yeo, S. W. Clinical experience of 71 cases of congenital middle ear cholesteatoma. *Acta Otolaryngol* 2010;**130**(1): 62-67.

15. Crowson MGR, V. H. Chapurin, N. Cunningham, C. D., 3rd Kaylie, D. M. Cost analysis and outcomes of a second-look tympanoplasty-mastoidectomy strategy for cholesteatoma. *The Laryngoscope* 2016;**126**(11): 2574-2579.

16. Danesi GC, Timothy Panciera, Davide Thomas Manni, Vito Côté, David WJ. Sanna classification and prognosis of cholesteatoma of the petrous part of the temporal bone: a retrospective series of 81 patients. *Otology & Neurotology* 2016;**37**(6): 787-792.

17. Darrouzet VD, J. Y. Portmann, D. Bebear, J. P. Preference for the closed technique in the management of cholesteatoma of the middle ear in children: a retrospective study of 215 consecutive patients treated over 10 years. *The American journal of otology* 2000;**21**(4): 474-481.

18. Das SD, M. Panja, T. Sinha, R. Chronic Draining Ear and Cholesteatoma Recidivism: A Retrospection from Clinical, Imaging, and Surgical Perspectives. *Turkish archives of otorhinolaryngology* 2019;**57**(3): 133-139.

19. De Corso EM, M. R. Scarano, E. Paludetti, G. Aural acquired cholesteatoma in children: surgical findings, recurrence and functional results. *Int J Pediatr Otorhinolaryngol* 2006;**70**(7): 1269-1273.

20. DeRowe AS, Gidon Fishman, Gadi Berco, Eli Avraham, Shabtai Landsberg, Roee Sadé, Jacob. Long-term outcome of atticotomy for cholesteatoma in children. *Otology & Neurotology* 2005;**26**(3): 472-475.

21. Diom ESC, Z. Tall, A. Ndiaye, M. Pegbessou, E. Ndiaye, I. C. Diallo, B. K. Diouf, R. Diop, E. M. Management of acquired cholesteatoma in children: A 15 year review in ENT service of CHNU de FANN Dakar. *International Journal of Pediatric Otorhinolaryngology* 2013;**77**(12): 1998-2003.

22. Edfeldt LK, A. Strömbäck, K. Köbler, S. Rask-Andersen, H. Surgical treatment of paediatric cholesteatoma: long-term follow up in comparison with adults. *Int J Pediatr Otorhinolaryngol* 2012;**76**(8): 1091-1097.

23. Edfeldt LS, K. Kinnefors, A. Rask-Andersen, H. Surgical treatment of adult cholesteatoma: long-term follow-up using total reconstruction procedure without staging. *Acta Otolaryngol* 2013;**133**(1): 28-34.

24. Erfurt CW, Sanne F Straatman, Louise V Smit, Adriana L Stokroos, Robert J Thomeer, Hans GXM. Canal-wall up cholesteatoma surgery with mastoid obliteration leads to lower rates of disease recurrence without affecting hearing outcomes. *Frontiers in surgery* 2024;**11**: 1381481.

25. Ferlito SLM, I. Merlino, F. Cocuzza, S. Di Stadio, A. Cammaroto, G. Bartel, R. Fadda, G. Iannella, G. Mat, Q. Gargula, S. Michel, J. Fakhry, N. Maniaci, A. Long-Term Anatomical and Hearing Outcomes of Canal Wall down Tympanoplasty for Tympano-Mastoid Cholesteatoma: A 20-Year Retrospective Study. *Life (Basel, Switzerland)* 2022;**12**(11).

26. Glikson EF, G. Sagiv, D. Wolf, M. Migirov, L. Shapira, Y. Trans-canal endoscopic ear surgery and canal wall-up tympano-mastoidectomy for pediatric middle ear cholesteatoma. *European archives of oto-rhino-laryngology : official journal of the European Federation of Oto-Rhino-Laryngological Societies (EUFOS) : affiliated with the German Society for Oto-Rhino-Laryngology - Head and Neck Surgery* 2019;**276**(11): 3021-3026.

27. Govil NS, Amanda L Georg, Matthew W Yellon, Robert F. The role of tympanostomy tubes in surgery for acquired retraction pocket cholesteatoma. *International Journal of Pediatric Otorhinolaryngology* 2015;**79**(12).

28. Hatano MI, M. Yoshizaki, T. Retrograde mastoidectomy on demand with soft-wall reconstruction in pediatric cholesteatoma. *Acta Otolaryngol* 2010;**130**(10): 1113-1118.

29. Hatano MI, M. Sugimoto, H. Noda, M. Hasegawa, H. Yoshizaki, T. Soft-wall reconstruction of the canal wall with retrograde bone work for pediatric cholesteatoma: Long-term results. *Int J Pediatr Otorhinolaryngol* 2016;**91**: 159-165.

30. Hellingman CAG, S. de Wolf, M. J. F. Ebbens, F. A. van Spronsen, E. Canal wall up surgery with mastoid and epitympanic obliteration in acquired cholesteatoma. *The Laryngoscope* 2019;**129**(4): 981-985.

31. Hou ZW, F. Zuo, W. Liu, Y. Wang, W. Hou, K. Jia, J. Yang, S. Application of a novel transcanal keyhole technique in endoscopic cholesteatoma surgery. *Acta Otolaryngol* 2021;**141**(4): 328-333.

32. Hu XC, M. Dai, W. Zhang, C. Li, S. Efficiency of intraoperative endoscopic inspection in reducing residuals in canal-wall-up surgery for pediatric cholesteatoma involving the mastoid. *European Archives of Oto-Rhino-Laryngology* 2023;**280**(8): 3593-3600.

33. Ikeda MY, Shinya Ikui, Akihiro Shigihara, Shuntaro. Canal wall down tympanoplasty with canal reconstruction for middle-ear cholesteatoma: post-operative hearing, cholesteatoma recurrence, and status of re-aeration of reconstructed middle-ear cavity. *The Journal of Laryngology & Otology* 2003;**117**(4): 249-255.

34. Inanli SÖ, Ö Batman, Ç Tutkun, A. Üneri, C. Sehitoglu, M. A. Clinical importance of supratubal recess in cholesteatoma surgery. *OTOLOGY & NEUROTOLOGY* 2001;**22**(6): 754-760.

35. James AL. Cholesteatoma Severity Determines the Risk of Recurrent Paediatric Cholesteatoma More Than the Surgical Approach. *Journal of Clinical Medicine* 2024;**13**(3): 836.

36. Jenks CMP, Patricia L Federici, Gaia Villari, Domenico Presutti, Livio James, Adrian L Hoff, Stephen R. Transcanal endoscopic ear surgery for congenital cholesteatoma: a multi-institutional series. *Otolaryngology–Head and Neck Surgery* 2022;**167**(3): 537-544.

37. Killeen DET, A. M. Kou, Y. F. Kutz, J. W., Jr. Isaacson, B. Recidivism After Endoscopic Treatment of Cholesteatoma. *Otology & neurotology : official publication of the American Otological Society, American Neurotology Society [and] European Academy of Otology and Neurotology* 2019;**40**(10): 1313-1321.

38. Kim JHC, S. H. Chung, J. W. Clinical results of atticoantrotomy with attic reconstruction or attic obliteration for patients with an attic cholesteatoma. *Clinical and experimental otorhinolaryngology* 2009;**2**(1): 39-43.

39. Komori MY, N. Hyodo, J. Minoda, R. Hinohira, Y. Five-year postoperative outcomes of modified staged canal wall up tympanoplasty for primary acquired cholesteatoma. *European archives of oto-rhino-laryngology : official journal of the European Federation of Oto-Rhino-Laryngological Societies (EUFOS) : affiliated with the German Society for Oto-Rhino-Laryngology - Head and Neck Surgery* 2018;**275**(3): 691-698.

40. Komori MM, Y. Tono, T. Matsuda, K. Yamamoto, Y. Sakagami, M. Hato, N. Kojima, H. Takahashi, H. Nationwide survey of middle ear cholesteatoma surgery cases in Japan: Results from the Japan Otological society registry using the JOS staging and classification system * , **. *AURIS NASUS LARYNX* 2021;**48**(4): 555-564.

41. Kuo C-LS, An-Suey Liao, Wen-Huei Ho, Ching-Yin Lien, Chiang-Feng. Can long-term hearing preservation be expected in children following cholesteatoma surgery? Results from a 14-year-long study of atticotomy-limited mastoidectomy with cartilage reconstruction. *Audiology and Neurotology* 2012;**17**(6): 386-394.

42. Lazard DSR, Gilles Denoyelle, Françoise Chauvin, Pierre Garabédian, Erea‐Noel. Congenital cholesteatoma: risk factors for residual disease and retraction pockets—a report on 117 cases. *The Laryngoscope* 2007;**117**(4): 634-637.

43. Lee JHH, S. M. Kim, C. W. Park, Y. H. Baek, S. H. Attic cholesteatoma with tiny retraction of pars flaccida. *Auris Nasus Larynx* 2015;**42**(2): 107-112.

44. Manzoor NFT, Douglas J McLeod, Megan E Sherry, Alexander D Perkins, Elizabeth L Haynes, David S Rivas, Alejandro. Comparative Analysis of Recidivism After Endoscopic and Microscopic-Based Cholesteatoma Resection. *Otology & Neurotology* 2022;**43**(4): 466-471.

45. Marchioni DV, D. Mattioli, F. Alicandri-Ciufelli, M. Piccinini, A. Presutti, L. Endoscopic Management of Attic Cholesteatoma A Single-Institution Experience. *OTOLARYNGOLOGIC CLINICS OF NORTH AMERICA* 2013;**46**(2): 201-+.

46. Marchioni DS, D. Rubini, A. Villari, D. Genovese, E. Artioli, F. Presutti, L. Endoscopic exclusive transcanal approach to the tympanic cavity cholesteatoma in pediatric patients: our experience. *Int J Pediatr Otorhinolaryngol* 2015;**79**(3): 316-322.

47. Minovi AV, J. Volkenstein, S. Dornhoffer, J. Dazert, S. Functional results after cholesteatoma surgery in an adult population using the retrograde mastoidectomy technique. *European archives of oto-rhino-laryngology : official journal of the European Federation of Oto-Rhino-Laryngological Societies (EUFOS) : affiliated with the German Society for Oto-Rhino-Laryngology - Head and Neck Surgery* 2014;**271**(3): 495-501.

48. Mishiro YS, M. Kitahara, T. Kondoh, K. Okumura, S. The investigation of the recurrence rate of cholesteatoma using Kaplan-Meier survival analysis. *Otology & neurotology : official publication of the American Otological Society, American Neurotology Society [and] European Academy of Otology and Neurotology* 2008;**29**(6): 803-806.

49. Mizutari KT, S. Kimura, E. Inuzuka, E. Shiotani, A. Patency of Anterior Epitympanic Space and Surgical Outcomes After Endoscopic Ear Surgery for the Attic Cholesteatoma. *Otology & neurotology : official publication of the American Otological Society, American Neurotology Society [and] European Academy of Otology and Neurotology* 2021;**42**(2): 266-273.

50. Møller PRP, Christina Nygaard Grosfjeld, Line R Faber, Christian E Djurhuus, Bjarki D. Recurrence of cholesteatoma-a retrospective study including 1,006 patients for more than 33 years. *International Archives of Otorhinolaryngology* 2020;**24**(01): e18-e23.

51. Morita YY, Y. Oshima, S. Takahashi, K. Takahashi, S. Acquired cholesteatoma in children: clinical features and surgical outcome. *Auris Nasus Larynx* 2014;**41**(5): 417-421.

52. Morita YT, K. Izumi, S. Kubota, Y. Ohshima, S. Yamamoto, Y. Takahashi, S. Horii, A. Risk factors of recurrence in pediatric congenital cholesteatoma. In: Otology and Neurotology; 2017; 2017. p. 1463-1469.

53. Motegi MY, Y. Ouchi, K. Akutsu, T. Tada, T. Kurihara, S. Takahashi, M. Sampei, S. Sano, H. Morino, T. Komori, M. Yamamoto, K. Sakurai, Y. Kojima, H. The impact of middle ear aeration on surgical outcome after intact canal wall tympanoplasty for cholesteatoma. *Auris Nasus Larynx* 2020;**47**(6): 965-975.

54. Myers ENS, Sven-Eric Drozdziewicz, Dominika Tos, Mirko Hougaard-Jensen, Annette. Recurrence of attic cholesteatoma: different methods of estimating recurrence rates. *Otolaryngology—Head and Neck Surgery* 2000;**123**(3): 283-287.

55. Nassif NdZ, L. O. R. The Role of Endoscopic Assistance in Surgery for Pediatric Cholesteatoma in Reducing Residual and Recurrent Disease. *CHILDREN-BASEL* 2024;**11**(3).

56. Neudert ML, S. Lasurashvili, N. Kemper, M. Beleites, T. Zahnert, T. Cholesteatoma recidivism: comparison of three different surgical techniques. *Otology & neurotology : official publication of the American Otological Society, American Neurotology Society [and] European Academy of Otology and Neurotology* 2014;**35**(10): 1801-1808.

57. Pareschi RL, D Nucci, R. Canal wall down approach for tympano-mastoid cholesteatoma: long-term results and prognostic factors. *Acta Otorhinolaryngologica Italica* 2019;**39**(2): 122.

58. Park K-HP, Shi-Nae Chang, Ki-Hong Jung, Min-Kyo Yeo, Sang-Won. Congenital middle ear cholesteatoma in children; retrospective review of 35 cases. *Journal of Korean medical science* 2009;**24**(1): 126.

59. Park KTS, Jae-Jin Moon, Sung Joong Lee, Jun Ho Chang, Sun O Oh, Seung Ha. Choice of approach for revision surgery in cases with recurring chronic otitis media with cholesteatoma after the canal wall up procedure. *Auris Nasus Larynx* 2011;**38**(2): 190-195.

60. Piras GS, V. Taibah, A. Russo, A. Caruso, A. Grinblat, G. Sanna, M. Long term outcomes of canal wall up and canal wall down tympanomastoidectomies in pediatric cholesteatoma. *Int J Pediatr Otorhinolaryngol* 2021;**150**: 110887.

61. Prasad SCLM, C. Medina, M. Vincenti, V. Bacciu, A. Bacciu, S. Pasanisi, E. Long-term surgical and functional outcomes of the intact canal wall technique for middle ear cholesteatoma in the paediatric population. *Acta otorhinolaryngologica Italica : organo ufficiale della Societa italiana di otorinolaringologia e chirurgia cervico-facciale* 2014;**34**(5): 354-361.

62. Presutti LA, Lukas Rubini, Alessia Ruberto, Marco Alicandri-Ciufelli, Matteo Dematte, Marco Caversaccio, Marco Marchioni, Daniele. The impact of the transcanal endoscopic approach and mastoid preservation on recurrence of primary acquired attic cholesteatoma. *Otology & Neurotology* 2018;**39**(4): 445-450.

63. Qotb MF, T. Ragab, W. Single Stage Canal Wall Down Mastoidectomy with Reconstruction of the Canal Wall: 5 Years' Experience in Fayoum Province, Egypt. *The journal of international advanced otology* 2017;**13**(2): 181-185.

64. Quérat CM, C. Prades, J. M. Richard, C. Canal wall up tympanoplasty for cholesteatoma with intact stapes. Comparison of hearing results between cartilage and PORP on stapes and impact of malleus removal and total reinforcement of the tympanic membrane by cartilage. *Eur Ann Otorhinolaryngol Head Neck Dis* 2014;**131**(4): 211-216.

65. Reddy TND, S. N. Shetty, A. Maini, S. Transcanal atticoaditotomy and transcortical mastoidectomy for cholesteatoma: the Farrior-Olaizola technique revisited. *The Annals of otology, rhinology, and laryngology* 2001;**110**(8): 739-745.

66. Roth TNZ, P. Haeusler, R. Caversaccio, M. D. Cholesteatoma surgery in children: long-term results of the inside-out technique. *Int J Pediatr Otorhinolaryngol* 2013;**77**(5): 843-846.

67. Roux AB, D. Lescanne, E. Cottier, J. P. Robier, A. Canal wall reconstruction in cholesteatoma surgeries: rate of residual. *European archives of oto-rhino-laryngology : official journal of the European Federation of Oto-Rhino-Laryngological Societies (EUFOS) : affiliated with the German Society for Oto-Rhino-Laryngology - Head and Neck Surgery* 2015;**272**(10): 2791-2797.

68. Schraff SAS, Barry. Pediatric cholesteatoma: a retrospective review. *International journal of pediatric otorhinolaryngology* 2006;**70**(3): 385-393.

69. Shin CHK, Woo Seok Park, Hong Ju Chung, Jong Woo Ahn, Joong Ho. Is there a clinical difference in paediatric congenital cholesteatoma according to age? *The Journal of Laryngology & Otology* 2023;**137**(6): 643-650.

70. Silvola JP, T. One-stage revision surgery for pediatric cholesteatoma: long-term results and comparison with primary surgery. *Int J Pediatr Otorhinolaryngol* 2000;**56**(2): 135-139.

71. Song ISH, Won Gue Lim, Kang Hyeon Nam, Kuk Jin Yoo, Myung Hoon Rah, Yoon Chan Choi, June. Clinical characteristics and treatment outcomes of congenital cholesteatoma. *The journal of international advanced otology* 2019;**15**(3): 386.

72. Sun JQS, J. W. Hu, Y. M. Lv, Q. P. Wang, Y. F. Li, X. G. Xu, W. Q. Wang, S. J. Mei, Z. F. Wan, G. L. Canal wall-down mastoidectomy with mastoid obliteration for pediatric cholesteatoma. *ACTA OTO-LARYNGOLOGICA* 2010;**130**(2): 259-262.

73. Trinidade AS, A. Yung, M. W. Pediatric cholesteatoma surgery using a single-staged canal wall down approach: results of a 5-year longitudinal study. *Otology & neurotology : official publication of the American Otological Society, American Neurotology Society [and] European Academy of Otology and Neurotology* 2015;**36**(1): 82-85.

74. van Dinther JJSV, J. P. Camp, S. De Foer, B. Casselman, J. Somers, T. Zarowski, A. Cremers, Cwrj Offeciers, E. The Bony Obliteration Tympanoplasty in Pediatric Cholesteatoma: Long-term Safety and Hygienic Results. *OTOLOGY & NEUROTOLOGY* 2015;**36**(9): 1504-1509.

75. van Waegeningh HFvD, J. J. S. Vanspauwen, R. Zarowski, A. Offeciers, E. The bony obliteration tympanoplasty in cholesteatoma: safety, hygiene and hearing outcome: allograft versus autograft tympanic membrane reconstruction. *European archives of oto-rhino-laryngology : official journal of the European Federation of Oto-Rhino-Laryngological Societies (EUFOS) : affiliated with the German Society for Oto-Rhino-Laryngology - Head and Neck Surgery* 2021;**278**(6): 1805-1813.

76. Vartiainen E. Ten-year results of canal wall down mastoidectomy for acquired cholesteatoma. *Auris Nasus Larynx* 2000;**27**(3): 227-229.

77. Visvanathan VK, H. Morrissey, M. S. Cholesteatoma surgery in children: 10-year retrospective review. *The Journal of laryngology and otology* 2012;**126**(5): 450-453.

78. Walker PCM, S. E. Hansen, M. R. Gantz, B. J. Long-term results of canal wall reconstruction tympanomastoidectomy. *Otology & neurotology : official publication of the American Otological Society, American Neurotology Society [and] European Academy of Otology and Neurotology* 2014;**35**(1): e24-30.

79. Wilson KFH, R. N. Shelton, C. Tympanoplasty with intact canal wall mastoidectomy for cholesteatoma: long-term surgical outcomes. *Otolaryngology--head and neck surgery : official journal of American Academy of Otolaryngology-Head and Neck Surgery* 2013;**149**(2): 292-295.

80. Wu NZ, W. Wang, F. Hou, K. Yang, S. Hou, Z. Scutum reconstruction technique and classification in endoscopic middle ear cholesteatoma surgery. *Acta Oto-Laryngologica* 2020: 904-908.

81. Yamamoto YT, K. Morita, Y. Ohshima, S. Takahashi, S. Long-term follow-up results of canal wall down tympanoplasty with mastoid obliteration using the bone pate plate for canal wall reconstruction in cholesteatoma surgery. *Otology & neurotology : official publication of the American Otological Society, American Neurotology Society [and] European Academy of Otology and Neurotology* 2014;**35**(6): 961-965.

82. Yang WYW, C. Recurrence rate of cholesteatoma with autoclaved incus autograft. *Otology & neurotology : official publication of the American Otological Society, American Neurotology Society [and] European Academy of Otology and Neurotology* 2014;**35**(10): e307-309.

83. Yung MJ, N. L. Vowler, S. L. A 5-year observational study of the outcome in pediatric cholesteatoma surgery. *Otology & neurotology : official publication of the American Otological Society, American Neurotology Society [and] European Academy of Otology and Neurotology* 2007;**28**(8): 1038-1040.

84. Zanetti DDL, F. Negri, M. Vincenti, V. Surgical management of middle ear cholesteatoma in children with Turner syndrome: a multicenter experience. *Acta bio-medica : Atenei Parmensis* 2018;**89**(3): 382-388.
